# Abstracts and Gleanings

**Published:** 1886-10-20

**Authors:** 


### ABSTRACTS AND GLEANINGS.

**Varicocele.**—I have preserved notes of six patients so operated on, the earliest in January, 1878. They were all young men, the subjects of advanced varicocele on the left side, and there existed softening of the testicle in two of them. In all the operation was confined to the left side, and was done in the same manner save that in one of them anæsthesia was not employed. They suffered from no fever or constitutional disturbance after the operation; the local reaction was confined to the formation of a firm induration at the site of operation, which, in the course of a few months, slowly disappeared. The result was in every instance satisfactory at the time, and most of them I have seen or heard from long after the operation, and they have found the cure complete and permanent.

The operation was carried out as follows: The patient was, in all cases save one, put under the influence of chloroform or ether in bed, and the scrotum disinfected by a five-per cent. solution of carbolic acid. The patient, who was not anæsthetized, was operated while sitting on a chair. After disinfection the left half of the scrotum was, by the usual maneuver, seized three quarters of an inch above the testicle between the forefinger and thumb of the left hand, and its contents allowed to slip back and escape until the cord-like vas deferns had slipped out of grasp. At this point the finger and thumb squeezed the skin of the two sides of the scrotum together, to squeeze the veins away from the just-escaped vas, and a threaded needle was thrust through the scrotum, at this spot. A handled needle with a large eye at its point was employed, and its thread was the strongest surgeon's silk, disinfected either by having been boiled in five-per cent. carbolic solution or by Kocher's method of twenty-four hour's soaking in German oil of juniper, the thread being afterward kept in absolute alcohol. The needle was disinfected by being washed first with oil of turpentine and then with carbolic lotion. Care was had in thrusting the needle through the scrotum to avoid, at the points both of entrance and emergence of the needle, the tubular sebaceous scrotal glands from which the hairs emerge, as they are always full of bacteria and their disinfection is an impossibility. The needle was then unthreaded and withdrawn, leaving the thread in the track. The skin of the front of the scrotum was then seized by the left forefinger and thumb and drawn foward in a fold between them until the punctures from which the thread emerged were drawn forward over the dilated veins to the base of the folds. They were there squeezed together and steadied by the finger and thumb, and the needle, this time without any thread, was once more passed through the scrotum, entering and emerging by the same points as before. The end of the thread emerging beside the needle point was threaded into its eye, and the needle was withdrawn, carrying the thread with it, so that both ends of the thread emerged by the same point where the needle having been detached, the long ends of the thread were tied by a surgical knot and tightened upon the veins and tissues they embraced with the utmost strength that could be applied. A triple knot was made, the ends of the silk were cut off short and the knot permitted to sink into the depth of the scrotum. The puckerings inward of the needle apertures, due to the first and second needle tracks not quite coinciding in the subcutaneous tissues, were freed by pulling the skin outward at these spots until the included fibres gave away and allowed the skin to fall into its natural position entirely unconnected with the knot.

Another exactly similar operation was made an inch (or two finger breadths, as the case required) higher up the veins, and the operation was then complete.

The scrotum was again disinfected, surrounded by a sheet of salicylic wool, and the patient laid in bed with the testes elevated.

One of my patients submitted to the operation without anæsthesia, but as a rule it is sufficiently painful to demand the administration of an anæsthetic, and the careful carrying out of the disinfection and necessary steps are much facilitated thereby.

A knot the size of the point of the thumb appears between and around the ligatured points, and a slight degree of scrotal edema can be detected, lasting for a few days. The patient suffers little pain, and as the needle punctures are agglutinated at once, frequent dressing is needless.

A daily renewal of the salicylic wool during the first three days is desirable, after that no further dressing is required. The knot at the site of the operation slowly disappears, and at the end of three weeks the patient can safely walk about, using, however, a suspensory bandage, and being careful to avoid strain, pressure, or fatigue of the part.

The disappearance of the last traces of the knot demands a month or two for its accomplishment, but eventually no trace remains of the operation having been performed.—*Alex. Ogston, C. M. Annals of Surgery, August, 1886.*

**Ophthalmology in the London Ophthalmic Hospital.**—Dr. W. C. Still, writing from this Hospital (*Nashville Medical Journal*) says: I have seen Mr. Lang, a young and aspiring oculist connected with this hospital, perform two or three times what is known as "Mules's operation," introduced by Dr. Mules, of Manchester, Eng. It is for those cases in which it is decided to sacrifice the eye to prevent sympathetic trouble in the other. The object of this special operation is, as you doubtless know, to leave a more suitable stump for carrying an artificial eye than results after the ordinary operation of excision, or even after abscission. An artificial vitreous is substituted for the one removed. The operation is thus performed: The cornea is removed at the sclero-corneal junction, and the contents of the globe removed as in ulceration, care being taken to remove every particle of the uveal tract. It is very important to do this latter thoroughly, as any remains of the uvea is liable to set up sympathetic trouble in the other eye. The sclera, with all its external attachments of nerves and muscles, remains intact. The edge of the sclera is then nicked at one point sufficiently to allow the insertion of the "artificial vitreous," a hollow glass sphere of suitable size. It should not be quite large enough to completely fill the sclerotic cavity, as stretching might cause sloughing of the sclera, which is one of the "accidents" of this operation which sometimes occur. The globe is not inserted until bleeding has about ceased. Then the sclera is first stitched over the globe, and finally the conjunctiva. If the operation "succeeds" you have a globe of almost natural size, with almost perfect movements, on which an artificial eye can rest.

The advantages claimed for the operation are obvious enough, namely, a comparatively full-size globe is secured, thus preventing the shrunken appearance so general after ordinary excission, and the attachments of the muscles to the sclerotic being preserved intact, the eye retains all the movement of the normal eye.

Mr. Lang exhibited a case—a young woman—at a recent meeting of the Ophthalomical Society of the United Kingdom, which I had the pleasure of attending. If the iris had had the proper tint, the eye would have almost defied detection.

Another oculist, whose name I do not now recall, showed a case in which a silver, instead of a glass, globe had been used. He made the point that silver is as innocuous as glass, and not liable to be broken *in situ* as glass. On the other hand, it is held that the glass globes are specially tempered, so as to be difficult to break, especially when lying on the soft cushion of pus and ocular fat. Of course the operation is at present on trial, and it is objected that, as the optic and ciliary nerves and ocular lymphatics are still present, the danger of subsequent sympathetic inflammation in the other eye remains.

Why not perform optico-ciliary neurotomy at the same time, or as a preliminary operation? I have not seen it suggested, but it seems to me that it would combine the good points of both operations.

Another new operation done here is one suggested by some German writer. It is the removal of the strip of palpebral conjunctiva which contains the so-called "granulations" in old and obstinate cases of trachoma. The edges of the remaining conjunctiva are then stitched together. The German "inventor" of this operation claims rapid and brilliant results for this operation.

I mention one more point, and that probably the most important one. I may call it Prof. John Tweedy's modification of Græfe's cataract operation. This definition may not be very accurate, but I will try to supply in a brief description what is lacking in my definition. He makes the initial puncture and counter-puncture in the sclera one or two milimetres posterior to the sclero-corneal junction, and brings the knife out about the same distance anterior to said line, seeking to strike a "happy mein" between the all-sclera and the all-corneal incisions, thus gaining a firm sclerotic base without interfering so much with the corneal nutrition supply as an all-sclera, or even a limbus, incision would. But what he considers as much more important, and which is more especially his own modification, is his method of extracting the lens.

After doing the second stage—the iridectomy—he again takes the Græfe knife, and, instead of lacerating the interior surface of the lens capsule, he makes with the point of the knife a peripheral section of said capsule at the edge corresponding with the iridectomy. Then, with a little to-and-fro stroking of the cornea below, the lens is extracted from its capsule, something as we slip on ophthalmoscopic "object" lenses from the little chamois pockets in which they are usually carried. The aqueous fluid escaping allows the cornea to fall into close contact with the unbroken anterior surface of the capsule, thus almost practically closing all entrance to septic particles except that into the capsule, and the capsule is more or less a dialyzing membrane. This method also prevents the escape of soft lens matter into the anterior chamber, where, as we know, it is liable to set up iritis, etc. The to-and-fro stroking of the cataract moves the lens in its capsule, thus detaching from the inner side of the capsule as much as practicable of the soft lens matter lying between the firmer central portion of the lens and the capsule, and which follows the lens as it is extended, or it is coaxed out afterward, at least as much of it as can be gotten out without doing any violence. As this soft lens material is safely inclosed in the capsule, where it can do no harm, he is not so very anxious to get it all out. In many cases, after any operation the inner layer of the capsule go proliferating soft lens material for awhile, and this operation compels this process to go on where it can do the least mischief, which cannot be said of those operations in which the front of the capsule is freely ruptured.

He asserts that he does not have to do secondary needlings (for opaque capsule) oftener than those who lacerate the anterior face of the capsule, and that when he does the capsule is in a practically normal condition, and a mere touch with a needle is sufficient to produce the required rent, while capsules which have been lacerated as in the ordinary operation are tough and often difficult to needle successfully.

He is positive that quite immature cataracts can be thus extracted with little if any more risk than in the ordinary "mature" cases, which would have been quite risky with the usual method of treating the lens. His rule is to operate whenever a cataract seriously interferes with a man's business, whether it is "ripe" or not. This certainly is a long step forward, as under the old rule persons have to wait for months and years for the cataract to "ripen," while, at the same time, they are deprived of all useful vision.

While Mr. Tweedy rightly declines to contend for any "priority" for any of these points, he can justly claim originality for them, at least for his method of opening the capsule, for he had been doing the operation for years before he knew of any one else doing it. Two years ago some one told him that Knapp, of New York, did it, and two months ago I heard a gentleman tell him that some one in Austria did it. There may be many others, as he says, but these are the only two of whom he has heard as performing it, and that several years after he had been regularly performing it.

**Urethan Ethyl Carbonate as a Physological Antidote to Strychnine.**—Some very interesting observations have quite recently been made by Professor Coze on the subject of the physiological effects of urethan, and more particularly on its antagonism to those of strychnine. The first point is its extreme tolerance when injected subcutaneously, or into the peritoneal cavity. Eight to ten grains so injected did not set up any local irritation in the frog, nor did thirty grains in the guinea-pig. No symptoms of gastric irritation were produced by from 80 to 100 grains given by the mouth in rabbits. The effect of the drug on the blood appears to consist in an increase of the amount of oxygen (4 per cent.); and the nervous exhaustion which follows its use may perhaps, be attributable to the greater difficulty with which the blood parts with its oxygen in favor of nervous system. Its influence on the tetanus of strychnine-poisoning was shown in the following manner. A frog just weighing under an ounce was injected with 1-700 of a grain of sulphate of strychnine (the minimum fatal dose being 1-1500 of a grain, according to Falck); and as soon as tetanus declared itself, five grains of urethan were injected. Four minutes later the tetanus ceased, and the muscles became completely relaxed. The next day, to the Professor's astonishment, the animal was all right again. This experiment was repeated many times, with larger and smaller doses of urethran; the effects were not so lasting with doses of less than 5 grains, but, if the tetanus returned, it could be almost instantly arrested by a further injection. A mixture of strychnine and urethran injected together gave rise to no tetanic symptoms, but rather to muscular relaxation.

On the guinea pig, the effects obtained were the same, the animal being some-what less amenable to the action of strychnine, one-eightieth of a grain of the drug was injected simultaneously into two guinea pigs. A quarter of an hour later, both the animals being tetanized, one of them was injected (into the peritoneal cavity) with 15 grains of urethan; the convulsions ceased; the respiration coming down first to 72 and then 40 per minute. The other animal, the check experiment, succumbed in twenty minutes to the effects of the strychnine.

For a rabbit weighing two and a half pounds, the minimum fatal dose of strychnine is 1-120 of a grain. Accordingly, to one weighing three pounds, successive injections were administered, amounting to 1-40 of a grain of the sulphate of strychnine, rapidly followed by a violent attack of spasm; the animal leaped and fell down breathless, and in a state bordering on asphyxia. Fifty grains of urethan was then promptly introduced into the stomach, after a few artificial respirations. In the course of a few minutes, the tetanic rigidity began to lessen, first in the hinder part of the body, respiration became deeper, and the animal fell into a quiet sleep. Two hours later, the rabbit raised its head without any convulsion, and gradually recovered. The next day, the only symptoms was a little weakness in the hinder legs; and, on the next day but one, the animal had to all appearances completely recovered. The contrary experiment was also made, the urethanized animal being injected with 1-50 of a grain of sulphate of strychnine, without the production of tetanus. All the experiments were repeated a certain number of times, but always with the same result. In other experiments, he administered a mixture of fifty grains of urethan with 1-50 of a grain of strychnine, and no spasm or rigidity followed. He pushed the strychnine to 1-5 of a grain without any apparent effect; and even with ¼ of a grain, although a few spasms manifested themselves, the animal did not die. On a dog weighing twenty-five pounds, 1-5 of a grain of strychnine was counteracted with 75 grains of urethan, and in twenty minutes the animal got up, and walked away with some difficulty. The next day he was all right again.

Dr. Coze suggests that clinical use should be made of the drug in conditions attended with convulsions, and more especially in cases of tetanus.—*British Medical Journal.*

**Creasote a Specific for Erysipelas.**—What is a specific? It may be defined as a remedy, which by its mechanical or chemical properties destroys or neutralizes the exciting or primary cause of disease.

Time was, when the advocate of a specific was laughed at by the scientific world, but since it is known that so many forms of disease are the direct result of some kind of germ life, it is no longer a misnomer to call a medicine which will certainly, and always, destroy the germ which produces so many forms of disease, a specific.

In the light of this definition, founded upon the experience of forty years successful practice in treating this form of disease, with creasote, the writer is prepared to indorse the heading of this article. Having used all the different remedies ordinarily prescribed, they have long since been laid aside, and this one used in all forms of the disease, exclusive, and with uniform success.

In 1863 it was the writer's fortune to spend several weeks in a military hospital in Memphis, as a volunteer surgeon, under the direction of Doctor Lord. In conversation with him, the use of this article was mentioned, which appeared new to him, and a case was put under treatment with it, with such prompt, favorable results as to elicit his hearty commendation, and, at his suggestion, Surgeon-General Hammond was informed of it.

All injuries, of whatever kind, have been treated with dressings of this remedy, and where this has been done from the first to last, in no instance has there been an attack of erysipelas.

The usual manner of application was in solution of 6 to 20 drops to the ounce of water, keeping the parts covered with cloths constantly wet with it. In ulcers or wounds it may be used in the form of a poultice, by stirring ground elm into the solution. The strength to be regulated according to the virulence of the attack. Ordinarily, 10 drops to the ounce is strong enough for the cutaneous form of the disease, and in dressings for wounds or recent injuries. If the inflammation threatens to spread rapidly, it should be increased to 20 or more drops to the ounce of water.

The antiseptic properties of this remedy render it of additional value, as it will certainly destroy the tendency to unhealthy suppuration, and thus prevent septicemia.

In the treatment of hundreds of cases of erysipelas, but a single fatal case has occurred, and that one in an old and depraved system. In less violent attacks, no other remedy was used, but where constitutional treatment was indicated the usual appropriate tonics were prescribed.

There is no question in my mind but that creasote is as much a specific in erysipelas as quinine is in intermittent, and may be used with as much confidence.—*St. Louis Medical Journal.*

**Typhoid Fever.**—Dr. J. M. Covington, of Rockingham, N. C. (in the *Medical World*), writes: I would have been very glad to have had Dr. W. S. Cline, of Tonis Brook, Va., with me during the endemic of typhoid fever in this village in 1880, '81 and 1882. His success has been so remarkable that I would like to have seen the result of his treatment with some of my patients. My loss was 25 per cent., and the loss of other physicians in the village and neighborhood was equally as great. Every plan of treatment advised by good authority was tried. The acid treatment of Flint and Da Costa; the calomel treatment as advised by some recent writer; the carbolic acid and iodine treatment of Bartholow; and in many cases, I might say, no medicine was given. Those that received no medicine did quite as well as any.

With an experience of twenty years, the following, in my opinion, is the best treatment: No medication should be given except for prominent symptoms. In the beginning, if the tongue is coated and bowels constipated, I give 5 grains of calomel with 3 grains of bi-carbonate of soda. Should the fever be 104° F. or more, with hot and dry skin, my rule is to remove the clothing, wrap him up in a sheet, place him on the floor on which has been placed a quilt covered with rubber cloth, and then sprinkle him thoroughly with cold water for ten minutes. This should be repeated when the temperature reaches 104° F. In the intervals, the extremities are frequently sponged with equal parts of cold water and vinegar. Later on in the disease, when the patient is weaker, the skin still hot and dry, I prefer sponging with whisky, and every night have his entire body well greased with fat meat. I eschew all antiseptics, such as quinia, digitalis, antipyrin, etc.—they are so apt to interfere with digestion, and, in this way, do more harm than good. As to diet, I give, in beginng, milk diluted with lime water, in small quantities frequently repeated; later on, beef tea, eggnog, etc. The beef juice I prefer is obtained by expressing with a lemon-squeezer a select piece of beefsteak which has been broiled just enough to crust both surfaces. I usually commence with stimulants about the middle of the second week. The diarrhœa is best controlled by bismuth and opium. Restlessness is soothed by frequent sponging with cold water and vinegar, and an occasional Dover's powder. Delirium, when it occurs in the early part of the attack, is best treated by chloral, but later in disease morphia hypodermically is preferred. For typanitis, cold compresses to the abdomen, and injections of spirits of turpentine and asafœtida. I

have never seen any good effect from turpentine in this very dangerous symptom used in any other way. Bronchitis, a very common complication, is best treated by dry cupping and carbonate of ammonia. Intestinal hemorrhage, by absolute rest, cold compresses to the bowels, and hypodermic of morphia and ergotine. Severe headache, so common during the first week, is relieved by the bromides and chloral.

Bed-sores are prevented by frequently bathing the tender parts with brandy and camphor. Should the sores penetrate the integuments, an application of equal parts of balsam copaiba and castor oil is good.

In conclusion, good nursing, fresh air, suitable diet, and remedies to meet important symptoms as they are indicated, is the proper course to pursue.

**The Hypodermic Injection of Morphia in Controlling Convulsions in Children.**—Dr. J. N. Study reports (in *Indiana Medical Journal*) a case of convulsions in a child relieved by morphia hypodermically. "When called to see the child, it was in a severe tonic convulsion which had continued for thirty minutes without any tendency to relaxation. The child was at once placed in a warm bath for twenty minutes, and a solution of bromide potassium and hydrate of chloral administered freely at intervals of fifteen minutes. The above treatment was persisted in for two hours without any tendency to relaxation, when chloroform by inhalation was used with the effect of temporarily controlling the convulsion. But immediately upon its withdrawal the convulsion returned as severe as before using. I now decided, as the case had assumed a very serious nature, to inject hypodermically one-twenty-fourth of a grain of morphia which was followed in ten minutes by a general relaxation and complete arrest of the convulsion which had continued three hours. No dangerous symptoms followed its use, and there was no tendency for the convulsions to return, although the attack was followed by measles complicated with a severe broncho-pneumonia."

So far as I am aware there have been but few cases reported where morphia hypodermically had been used in convulsions of children. Dr. H. Plummer, of Harrodsburg, Ky., reports in *Medical Record*, of March 27th, a case where bromide of potassium in 5 grain doses failed to control the convulsions in an infant, aged twenty two months. He administered 1-6 of a grain of morphia hypodermically with the effect of controlling the convulsions, his patient falling into a sleep lasting twenty-four hours, awaking only to drink. The convulsions did not return."

"Dr. G. T. Fanning, of New York, reports in *Medical Record*, April 24th, two cases of two years and two and one-half years of age, where morphia hypodermically was administered in convulsions with ultimate good results."

"Dr. C. S. Scofield, of Boston, reports a case in the *Medical Record*, of June 29th, where an infant, aged eighteen months had been in convulsions for two hours. All remedies tried had been of no avail in contolling the attack when he administered 1-12 of a grain of morphia hypodermically which was repeated at the expiration of twenty minutes with no improvement; a third dose was given twenty minutes later. In one hour from the first injection the child was sleeping quietly. The following morning the child was apparently as well as usual.

"I have seen no mention in any treatise on diseases of children where the use of morphia hypodermically was recommended to control convulsions; but knowing that morphia used in this way has great power to control puerperal convulsions in many instances, why should not its action be equally as productive of good in infantile convulsions where the nerve centers are in the highest possible state of exciteability? I should not think it wise treatment to employ this means of controlling convulsions in any case, however, till other well-known remedies such as the bromide of potassium and hydrate of chloral, the warm bath and chloroform had been thoroughly tried. But when such remedies fail I believe we have in morphia hypodermically administered a ready and prompt means of controlling many cases of infantile convulsions that otherwise would terminate fatally. When morphia is administered to young children in this way it is certainly wise to begin with the smallest possible dose which will control the convulsion, and repeat as may seem advisable. It certainly should never be given without knowing the exact amount administered. I should doubt the propriety of administering the 1-6 of a grain in a young child, as was done in one of the cases reported; but it may be that children laboring under such a high state of nervous excitability tolerate larger doses of morphia than they would otherwise.

**Hot Baths, Hot Packs and Pilocarpine Compared.**—Dr. Zelenetski, of St. Petersburg, in order to examine the comparative effects of hot baths, pilocarpine, and hot wet-sheet packing on nephritis, treated the same patients on different days by each of these methods, observing the effects of the temperature, pulse, etc.—Fifty-seven observations were made on seven patients, who were as nearly as possible under identical conditions. Twenty-three baths, eighteen hypodermic injections of pilocarpine, and fifteen hot packs were given. The hot baths produced the greatest loss of weight, averaging 801 grammes, and the packing the least, averaging 94 grammes, pilocarpine producing effects of an intermediate character. Here the mean loss of weight was 514 grammes, 360 by perspiration and 208 by salivation. The temperature rose considerably after the baths. and even at the end of three hours was always above normal The packing caused it to fall at first; but after an hour it rose, and returned to its original height within three hours. With pilocarpine it was reduced for two hours, and then rose to normal. The pulse corresponded to the temperature with the baths, but became slower with both packs and pilocarpine. The patients expressed themselves as feeling the most improvement after the baths; the pilocarpine causing complications, such as headache and nausea, and, in one case, vomiting and collapse.—*Medical Times.*

**On Lobelia Inflata in Asthma.**—The employment of lobelia inflata against asthmatic disorders has of late become obsolete, owing to the alleged inertness of the drug in the mentioned affection. Dr. Nunes writes to the *Bulletin Gener. de Ther.*, No. 4, 1886, that he feels convinced that the failure of lobelia in asthma is caused solely by using the drug in too small doses. Trousseau and Pidoux prescribed 20 to 25 drops of the tincture three to four times daily, Dujardin Beaumetz 20 to 50 drops, Gubler 1 to 2 fʒ, and Torres Ilomen even 2½ fʒ with very satisfactory results. No other clinician has ever employed the drug in so large a dose as Ilomen for fear of causing untoward symptoms or even poisoning. Nunes's practice of exhibiting the tincture of lobelia in doses of ½ to 1 f℥, without having ever met with an accident, shows how little the above-mentioned fears are justified. In order to intensify the expectorant action of lobelia, Nunes combines it with benzoate of ammonia. His results was very encouraging. The following cases are alluded to in Nunes's communication:

A man of 56 years of age was suffering for a year from strong dyspnœa and coughing, interfering with his sleep. On auscultation whistling and bellows-sounds were heard. Heart and blood-vessels were normal. Nunes prescribed

℞. Tinct. lobeliæ .................................. fʒ iv,

Ammon. benz.................................. ℥ iiss,

Aquæ dest...................................... ℥ vii.

M. S.—A tablespoonful every two hours.

Later the quantity of lobelia was doubled; the patient recovered completely.

A man 24 years of age suffered for three years from dyspnœa and cough. The vascular system was normal, but the liver was enlarged. The latter condition was removed by giving the patient

℞. Calomel...................................... gr. viii,

Pulv. rad. rhei, }

Saccharx., } aa.............................. gr. vii.

S.—Take before retiring.

Later the following was prescribed:

℞. Tinct. lobelia................................ fʒ v,

Ammon. benz.................................. ʒ iii,

Aquæ dest...................................... ℥ vii.

M. S.—Tablespoonful every two hours.

After three days, cough and dyspnœa had decreased. Nunes then ordered:

℞. Terpine........................................ gtt. xv,

Mixt. gumm.................................... ʒ vii.

S.—Tablespoonful hourly.

As the condition of the patient grew worse, Nunes resorted to the dose of 1 f℥ of lobelia in 7 f℥ of water, as given to the first patient, whereupon the patient recovered.

In two other cases, Nunes obtained the same favorable results by equally large doses of the tincture of lobelia.—*Therapeutic Gazette.*

**Cocaine in Cataract Extraction.**—In the *Journal of the American Medical Association* (August 21, 1886), Dr. George E. Frothingham publishes some cases illustrating the safety of cocaine as an anæsthetic in cataract extraction, and summarizes his conclusions as follows:

1. Cocaine relieves the operator from the embarrassments during the operation for cataract that arise from vomiting; also from the agitation of his patient which results from excessive bronchial secretion, or stertorous breathing. These are often very troublesome when ether or chloroform is used.

2. The danger to the result which often arises from nausea and vomiting after the extraction, when other anæsthetics are employed, is very surely avoided when cocaine is selected as the anæsthetic agent and is properly used.

3. The danger arising from the depressing effect of cocaine upon the nutrition of the cornea is no greater than in cases where ether or chloroform are used. The depression of the circulation, which often arises from either of them, may affect very injuriously the corneal nutrition.

4. The disturbance of the circulation of the interior of the eye, and consequent danger of panophthalmitis from this cause, is probably less in using cocaine for this operation than in resorting to general anæsthesia.

5. The danger of sepsis and consequent panophthalmitis from the use of cocaine may be avoided by using only fresh solutions. —*Therapeutic Gazette.*

**Conception of Male Children at the Time of the Post-Menstrual Anæmia.**—Dr. Camillo Furst, of Graz, publishes in the *Archiv fur Gynæcologie*, 1886, No. xxviii. 1, p. 14, a contribution to the interesting and frequently-discussed question, When and how is the sex of the conception-product determined? In the first section of the paper, which treats of "the time and causes of the determination of the sex in general," Furst proposes certain maxims which, though not new, will interest our readers. According to the author we find a surplus of male conceptions in the working classes and country inhabitants as compared with the well-to-do people and the inhabitants of cities. Likewise, we can look for the surplus of male infants during hard times and the concomitant rise of food-prices, and before the ultimate extinction of a race. If a deficient nutrition of the procreators produces a surplus of male children, our author continues to argue, we can be certain that also the state of nutrition of the fecundated ovum, especially shortly after conception, will influence the sexual differentiation. And as after menstruation the vessels of the genital organs assume an ischæmatous character,—forming the so-called post-menstrual anæmia,—Furst concludes that conceptions taking place immediately or shortly after menstruation will give a surplus of males on account of a relatively bad nutrition of the fecundated ovum. To strengthen his theory the author utilizes the statements of women confined in maternities, who mostly with an astonishing certainty could remember the end of the last menstruation and the day of conception. The statistics of the mentioned institutions show a very considerable surplus of male children for the first four or five days following menstruation, and a surplus of female ones for the succeeding period.—*Therapeutic Gazette.*

**Cholera Infantum.**—Dr. John A. Henning, of Garnett, Kans. (in the *Medical World*), says: As the season is here for cholera infantum, I desire to call the attention of practitioners to the treatment of that form of this disease in which it commences in the brain, very frequently manifested by convulsions. Perhaps in a majority of cases the brain and nervous system are more or less affected, as heat is the cause which depresses the vital forces, and by reflex action congestion and inflammation of the bowels ensue. Formerly, those cases baffled my skill, but now in all such cases I commence with this prescription:

℞. Bromidi potassi..............................ʒ i,

Fl. ex. gelsemii..............................ʒ ss,

Aquæ..............................℥ iv. M.

Sig. Teaspoonful every two hours.

I continue to give this prescription as long as any brain trouble is manifested. This will stop the convulsions and reduce the temperature. In one, two or three days all the train of difficulties will be removed. In the mean time, I give such other remedies as seem to be indicated. If there is no brain difficulty, I do not use this remedy at all.

Last week I was called to a child, two years old, taken very suddenly with convulsions, high fever and frequent discharges. This remedy soon removed all nervous trouble, and the child soon recovered.

**On Revaccination.**—In an essay on the "Revaccination of Young Individuals," published by Dr. Jules Besnier in the *Revue Mensuelle des Maladies de l' Enfance* (February, 1886), this author establishes the following conclusions:

1. The number of successful revaccinations in young subjects revaccinated for the first time increases with the advancing years, and reaches its maximum at the period of fifteen to twenty years. In adults, revaccinations are less frequently successful than in young subjects.

2. Certain diseases favor a successful revaccination at certain periods of life. Among these are the affections of the typhoid type; the ordinary eruptive fevers of childhood, and the chronic affections of old age have no such influence.

3 In the subjects vaccinated at birth and not revaccinated, the predisposition for variola and vaccinia reaches its maximum in the ages of fifteen to twenty, and decreases gradually as age advances.

4. This fact is a stringent reason for the revaccination of all persons at the stated ages of adolescence.

5. Bovine lymph is by all means preferable to human lymph.

6. In absence of an epidemic of variola, the months of March and April (Easter holidays) are most suitable for revaccination of school children.—*Medical Times.*

**Carbolic Treatment of Hemorrhoids.**—The strength of the solution must be regulated by the nature of the case, and in my own practice varies from 5-per cent. to pure crystallized acid. In a large vascular prolapsing tumor, which is well defined and somewhat pedunculated, 5 drops of pure acid may be used with the expectation of producing a circumscribed slough which will result in a radical cure. A 33-per cent. solution under the same conditions will probably produce consolidation and shrinkage without a slough, but the injections will have to be repeated several times. A small tumor, which protrudes but slightly, is not pedunculated, and can be seen and felt as a mere prominence on the mucous membrane, may be cured by a single injection of a 5-per cent. solution, which will cause it to become hard and decidedly reduce its size, while an injection of a 50-per cent. solution might make considerable trouble, the remedy being too powerful for the disease. Guided by this principle, some experience will soon determine the choice of the solution. There is no arbitrary rule which can be applied to every case. As in any other surgical operation, some cases will be more satisfactory than others, and an occasional accident must be expected; but, on the whole, it seems to be the best method of treatment yet devised.—*N. Y. Med. Jour.*

**Substitute for Circumcision.**—Take a pair of self-holding forceps, whose blades expand when the handles are compressed; pass the point down to the bottom of the prepuce, between it and the glans. Be careful not to get it into the urethra. Then expand the blades forcibly until they are separated about half an inch, and withdraw the instrument. The effect will be to tear the mucous membrane, the skin stretching. You can then push back the prepuce, uncover the glans, and cleanse the cavity of smegma. If there be any adhesions between the glans and foreskin tear them up with the end of a probe. The prepuce must be retracted and the parts washed every day for a week; a soothing ointment being used if necessary.

The results of this operation are perfectly satisfactory. In the last five years we have performed it in many cases, and have never known it to fail. We have learned to look upon the old operation of circumscision as barbarous and unecessary. Not more than six drops of blood are lost in the dilatation, and retraction occurs to a proper extent. If cocaine be used, the operation is rendered perfect, being simple, easy, safe, painless, bloodless and efficient. The originator is Professor William S. Stewart.—*Medical World.*

**Cocaine to Diminish Labor Pains.**—At the meeting of the Association of German Physicians in Prague, on April 8, 1886, Fischel presented the record of five cases in which local application of cocaine were used to diminish the pain of labor. He quoted the brilliant results obtained by Doleris, in whose observations the drug was applied to the vaginal mucosa and the external genitals in acqueous solution, or in the form of a salve, the strength in either case being 4 per cent. The amount used was from 40 to 60 drops of the solution and 45 to 60 grains of the salve. In this manner Doleris induced practically painless delivery in thirteen out of fifteen primiparæ.

Fischel's results were less striking. Employing, as a rule, weaker cocaine solutions, he records one case of absolute painless delivery, two in which the pain greatly diminished by the application, and two in which the results were negative.

These observations, apart from the practical interest, tend to show that the seat of the labor "pains" is not in the uterus, but in the dilating cervix and the vagina.—*Wiener medicinische Presse.*

**Delivery During Hypnosis.**—A pregnant woman, aged twenty-six, was admitted into the obstetrical clinic of C. Braun, in Vienna. Dr. Pritzl who reports the case in the *Wiener Medical Wochschr.*, 1886, No. 21, says that it was accidently discovered that she could easily be brought into a hypnotic state. The hypnotic sleep in her case set in rapidly and had no bad consequences of any kind. When, therefore, during the delivery the labor pains became very severe, he concluded to put her into the hypnotic state. He easily succeeded in doing so. The labor remained vigorous, the pauses became longer, and abdominal pressure continued to act; at the same time the os of the uterus dilated well and the delivery was happily concluded. The placenta was then delivered into the vagina and removed by the hand. On awakening, the patient felt very strong and did not remember any pain.

A remarkable fact was that abdominal pressure was brought about by reflex, for the patient being totally unconscious, no action of hers could have excited the pressure. Very little blood was lost during the delivery.—*Indiana Medical Journal.*

**Jaborandi in Erysipelas.**—We have given jaborandi a trial in Erysipelas, as recommended in the *Medical World* recently.

Result: Never before in our professional life have we witnessed such a powerful effect for good in this disease from any remedy! The first dose dissipated all our fears for the patient's safety. While the disease showed a tendency to spread whenever the jaborandi was suspended, even jumping over a circle of flexile collodion (which will occur in bad cases), the moment the remedy was resumed, the disease was checked.

We found it advisable to combine digitalis and quinine with the jaborandi after a few days, as the patient's heart showed symptoms of weakness. Of three cases of facial erysipelas treated at the same time one died, and the second was double the time in recovering which our case required.

**Post-Partum Hemorrhage.**—Dr. McManus, of Oakland, Cal., says: On finding the surface pale, the extremities cold, with profuse hemorrhage, I at once inject hypodermically from 10 to 15 drops of Majendie's solution of morphine. This produces invariably, in a few moments, a flushed surface, warm extremities, and a diminished or entire cessation of the flow.—*Med. Times.*

